# Antitumor Potential of Sericite Treatment Mediated by Cell Cycle Arrest in Triple-Negative MDA-MB231 Breast Cancer Cells

**DOI:** 10.1155/2022/2885293

**Published:** 2022-09-25

**Authors:** Seonhee Kim, Harsha Nagar, Ikjun Lee, Su-Jeong Choi, Shuyu Piao, Byeong Hwa Jeon, Shin Kwang Kang, Hee-Jung Song, Cuk-Seong Kim

**Affiliations:** ^1^Department of Physiology and Medical Science, School of Medicine, Chungnam National University, Daejeon, Republic of Korea; ^2^Department of Thoracic and Cardiovascular Surgery, Chungnam National University Hospital, Chungnam National University School of Medicine, Daejeon, Republic of Korea; ^3^Department of Neurology, Chungnam National University College of Medicine and Sejong Hospital, Sejong, Republic of Korea

## Abstract

Breast cancer is the most common cancer and the leading cause of cancer-related mortality among females worldwide. Triple-negative breast cancer (TNBC) accounts for about 10–15% of all breast cancers and is usually more aggressive and has a poorer prognosis. Sericite has been known to have antitumor and immune-stimulatory effects. Although the chemopreventive potential of sericite has been demonstrated in other cancers, its molecular pathways in TNBC still require investigation. Thus, in the present study, the antitumor mechanism of sericite against MDA-MB231 breast cancer cells was examined *in vitro* and in an *in vivo* xenograft mouse model. Sericite treatment reduced cell proliferation and cell proliferation marker proliferating cell nuclear antigen (PCNA) in MDA-MB231 cells. It also decreased the total cell number and arrested cells in the G0/G1 phase of the cell cycle with an increase in the phosphorylation of P53 and upregulation of cell cycle regulatory proteins P21 and P16. In addition, sericite treatment also induced apoptosis signaling, which was evident by the upregulation of apoptotic protein markers cleaved caspases 3 and 9. A reduction in reactive oxygen species (ROS), NADPH oxidase 4 (NOX4), p22phox, and heat shock proteins (HSPs) was also observed. Similar results were obtained *in vivo* with significantly reduced tumor volume in sericite-administered mice. Collectively, these findings suggest that sericite has antitumor potential based on its property to induce cell cycle arrest and apoptotic cell death and therefore could serve as a potential therapeutic agent and crucial candidate in anticancer drug development for TNBC.

## 1. Introduction

Cancer is a severe life-threatening disease that ranks as a leading cause of death worldwide. Breast cancer is the most common type of cancer and also the leading cause of cancer-related mortality in women [1, 2]. Breast cancer incidence and mortality rates greatly depend on the socioeconomic backgrounds of countries, with incidence rates estimated at 54.4% and 31.3% in developed and developing countries, respectively. Breast cancer is categorized into four primary molecular subtypes: luminal A and B, human epidermal growth factor receptor 2 (HER2) overexpressed, and basal-like subtypes [3]. Triple-negative breast cancer (TNBC) is similar to the basal-like subtype of breast cancer, as there is a lack of expression of hormone receptors (estrogen receptor (ER) and progesterone receptor (PR)) and HER-2 gene amplification in TNBC.

For the management and treatment of breast cancer, radiation, surgical resection, and systemic therapy, including hormonal or endocrine therapy, targeted therapy, chemotherapy, or a combination of these therapies, have been used in breast cancer patients [4]. Unfortunately, for TNBC, conventional targeted treatment options and endocrine therapies are limited, and outcomes are substantially worse [5]. Therefore, chemotherapy remains the only systemic modality available for TNBC because these cells do not express ER or PR and lack HER-2 overexpression. However, there are several side effects associated with chemotherapy, which include weakness, nausea, reduced resistance to infections, vomiting, and hair loss. Therefore, developing a suitable strategy for TNBC treatment to increase patient survival and reduce side effects is essential.

Inorganic nanomaterials show great potential for clinical applications as they possess many unique characteristics. The intrinsic physicochemical characteristics of inorganic nanoparticles make them outstanding therapeutic agents for cancer treatment. Sericite is the name given to very fine, ragged grains and aggregates of white or colorless micas, typically made of muscovite. Sericite is highly refractive and is found in hydrothermally altered rocks [6]. Traditionally, sericite has been used to alleviate pain in the reproductive organs and to treat bleeding, dysentery, gastric diseases, and inflammation [7]. It has been shown previously that mica demonstrates chemopreventive potential against colorectal cancers by blocking the cell cycle and proliferation [8]. In addition, mica has the ability to stimulate immune responses against viral infection. Therefore, mica has been used as feed supplements for enhancing immune responses [9]. Heat shock proteins (HSPs) are present or induced in all living cells to protect them from high-temperature stress and have been extensively associated with various cancers and their behaviors [10]. They have various tumorigenic properties, including inhibition of apoptosis and senescence and promotion of angiogenesis, invasion, and metastasis [11]. Due to its vital role in tumorigenesis, inhibition of HSP activity has been accepted as a significant biological strategy for designing chemotherapeutics against cancer.

The need for more efficacious and innovative therapies for the treatment of TNBC prompted us to investigate the antitumor effect of sericite in MDA-MB231 cells, a triple-negative human breast carcinoma cell line, and in a xenograft mouse model. Thus, this study aimed to investigate if sericite may be utilized as an adjuvant to mainline cancer treatment and if it can be developed as a therapeutic anticancer agent in TNBC therapy.

## 2. Materials and Methods

### 2.1. Cell Culture and Cell Growth

Human breast epithelial cells (MCF10A, CRL-10317), triple positive breast cancer cells (BT474, HTB-20), ER+/PR+/HER2-breast cancer cells (MCF7, HTB-22), ER-/PR-/HER2+ breast cancer cells (SKBR3, HTB-30), and triple-negative breast cancer cells (BT549, HTB-122 and MDA-MB231, and HTB-26) were obtained from American Type Culture Collection (ATCC, VA, USA). These cells were cultured at 37°C with 5% CO_2_ according to the manufacturer's instructions. Sericite (Gumcheon Corp., Okcheon-gun, Chungcheongbuk-do, South Korea) was dissolved in complete media first at concentrations of 10 mg/ml and 3 mg/ml. These were serially diluted further to 1 mg/ml and 0.3 mg/ml, respectively; and all the concentrations were then directly treated in the cells for 24 h. Cell counting was performed using ADAM-MC Automatic Cell Counter (Digital Bio. Seoul, South Korea) that functions using the Propidium Iodide (PI) staining method of dead cell staining. After treatment with sericite, cell counting was performed according to the manufacturer's instructions. The initial doses of sericite for the first screening were selected based on previous publications [8, 9] and the final dose range was chosen after preliminary experiments were performed to decide the best suitable dose for all further experiments.

### 2.2. Mouse Xenograft Models

All animal studies were performed in the animal facility following the guidelines of the Institutional Animal Use and Care Committee at Chungnam National University (CNUH-018-A0024-1). The animal experiments complied with the ARRIVE (Animal Research: Reporting of In Vivo Experiments) guidelines for the use of experimental animals with the approval of the Chungnam National University. Female Balb/c nude mice (4 weeks) were purchased from OrientBio Inc (Gyeonggi-do, KOREA). Mice were maintained in a controlled environment (ambient temperature 22–24°C; humidity 50–60%; 12-h light/dark cycle). In order to generate subcutaneous tumors, mice were anesthetized using Avertin (250 mg/kg), and MDA-MB231 cells (1 × 10^7^ cells/mouse) mixed with Matrigel (Corning, 356230) were injected subcutaneously into the flanks of the mice. For cancer assessment, cancer length and width were measured daily with digital calipers, and the volumes were calculated using the following formula: (length *∗* width^2^/2). Sericite was dissolved in saline in the same manner as for the in vitro studies, and based on pilot studies, the same doses as the in vitro experiments were administered. Following the establishment of subcutaneous MDA-MB231 xenograft in mice 24 days after tumor cell injection, mice with the same cancer volume (mm^3^) were divided into three groups (saline, 1 mg/kg sericite, or 3 mg/kg sericite) and fed 100 *μ*l of sericite solution (1 mg/kg or 3 mg/kg) or 100 *μ*l of saline daily for 12 days by oral gavage, after which the mice were sacrificed by an overdose of Avertin and the tumors were dissected for further experiments.

### 2.3. Immunoblotting

The following antibodies were used: anti-p16 (sc-377412), anti-p21 (sc-6246), anti-p53 (sc-393031), and anti-GAPDH (sc-47724) (Santa Cruz Biotechnology, Santa Cruz, CA, USA); anti-P-p53 (S15) (9284S), anti-cleaved Cas9 (9509S), and anti-cleaved Cas3 (9664S) (Cell Signaling Technology, Danvers, MA, USA); anti-Shc (610878, BD Biosciences, Franklin Lakes, NJ, USA); anti-P-p66Shc (S36) (ALX-804-358) (Enzo Life Sciences, Inc., NY, USA), anti-MMP9 (MA5-15886, ThermoFisher), and anti-PCNA (PC 10, Sigma-Aldrich, USA). Immunoblotting of 30 *μ*g of whole-cell lysate or tissue homogenate was performed as described previously [12]. Briefly, MDA-MB231 cells and cancer tissues were harvested and lysed in RIPA buffer containing protease and phosphatase inhibitors for 30 min on ice. After centrifugation at 13,000 rpm for 10 min, the protein concentration of cell and tissue lysates were measured using a bicinchoninic acid (BCA) protein assay kit (iNtRON, cat. 21071). Equal amounts of protein per well were resolved in 10–15% sodium dodecyl sulfate-polyacrylamide gel electrophoresis (SDS-PAGE) and transferred onto a nitrocellulose membrane. The membranes were then washed with Tris-buffered saline (10 mM Tris, 150 mM NaCl) containing 0.1% Tween 20 (TBST) and blocked in TBST containing 5% bovine serum albumin Fraction V (Roche, Basel, Switzerland) followed by incubation in appropriate primary and secondary antibodies. The chemiluminescent signal was developed using Super Signal West Pico or Femto Substrate (Pierce Biotechnology, Rockford, IL, USA). Band densities were quantified on a Gel Doc 2000 Chemi Doc system using Quantity One software (Bio-Rad, Hercules, CA, USA). Values were normalized to *β*-actin (loading control).

### 2.4. Real-Time PCR

Total RNA was isolated from cells using TRIzol reagent (Thermo Fisher Scientific). Complementary DNA (cDNA) was generated from total RNA using the MAXIME RT Premix Kit (iNtRON Biotechnology, Gyeonggi, South Korea). Relative RNA expression levels were determined by PCR using a SYBR qPCR premix (Enzynomics, Daejeon, Republic of Korea). The primer sequences are provided in Table S1. A preincubation for 10 min at 95°C was followed by 40 amplification cycles: 10 sec at 95°C, 20 sec at 60°C, and 30 sec at 72°C. The melting curve for PCR product analysis was determined by rapid cooling down from 95°C to 65°C and incubation at 65°C for 15 sec before heating to 95°C. To normalize for equal mRNA/cDNA amounts, PCR reactions with target-specific and with GAPDH-specific primer sets were always run in parallel for each sample, and relative expression levels were determined by the 2^−ΔΔCt^ method.

### 2.5. Histological Analysis

After washing with phosphate-buffered saline, tumor tissues were fixed with 4% (w/v) paraformaldehyde and then embedded in paraffin. Paraffin sections were deparaffinized and rehydrated according to standard protocols and stained with hematoxylin-eosin (H&E). For immunohistochemistry staining, tumor tissues were stained with primary antibodies anti-MMP9 (diluted in 1 : 100; Cat.MA5-15886, ThermoFisher), anti-CD31 (diluted in 1 : 100; Cat.550274, BD science), and anti-PCNA (diluted in 1 : 100; PC 10; Sigma-Aldrich) overnight at 4°C. HRP-conjugated anti-rabbit and anti-mouse IgG was treated for 60 min at room temperature. The color was developed for 30 secs by incubation with 3,3′-diaminobenzidine (DAB). Sections were counterstained with hematoxylin and examined using a microscope (Motic, Richmond, BC, Canada) at 100x magnification.

### 2.6. CCK-8 Cell Proliferation Assay

Cells were treated with sericite for 24 h. Cell proliferation was measured using a CCK-8 kit (Dojindo, Japan) according to the manufacturer's instructions. Briefly, cells were washed with PBS and suspended in a growth medium, including CCK-8 reagent added at 1/50 the media volume. Cells were then incubated at 37°C for 1 h in the dark. Cell proliferation was measured at a wavelength of 450 nm.

### 2.7. TUNEL Assay

TUNEL assay was used to detect DNA fragmentation, such as apoptosis. Cells were treated with sericite for 24 h. After 24 h incubation, cells were washed twice with PBS, detached from the plate using trypsin, and collected in a 15 ml tube. These cells were fixed in 100% ethanol overnight at 4°C. TUNEL assay was performed according to the manufacturer's instructions (FITC Abcam, cat. ab66108). Stained cells were analyzed by flow cytometry for FITC using a NovoCyte flow cytometer as per the manufacturer's instructions (ACEA Biosciences, San Diego, CA, USA). Flow cytometry data were analyzed using NovoExpress software. Fluorescence images were captured using a fluorescence microscope (Zeiss Axio imager M1).

### 2.8. Flow Cytometry

Cells were treated with sericite for 24 h. The next day, cells were analyzed by flow cytometry for cell cycle using a NovoCyte flow cytometer as per the manufacturer's instructions. Cells were washed twice with PBS and fixed in 70% ethanol overnight at 4°C followed by staining with PI (5 *μ*g/ml) (Thermo Fisher, 00-6990-50) and RNase A (1 mg/ml) (Sigma-Aldrich, R875-100 mg) in 500 *μ*l PBS for 30 min at 37°C. Flow cytometry data were analyzed using NovoExpress software.

### 2.9. ROS Detection

Amplex Red (A22188, Invitrogen, USA) was used to detect intracellular ROS production. MDA-MB231 cells, MCF10A cells, and tumor tissues were treated with saline or sericite. The reaction mixture contained 50 *μ*M Amplex Red and 0.1 U/ml HRP in Krebs–Ringer phosphate (KRPG) buffer (145 mM NaCl, 5.7 mM sodium phosphate, 4.86 mM KCl, 0.54 mM CaCl2, 1.22 mM MgSO4, 5.5 mM glucose, pH 7.35). Each reaction had a volume of 100 *μ*l. Moreover, 20 *μ*l of 1.5 × 10^4^ cells suspended in KRPG buffer was added to the 100 *μ*l reaction mixture. Absorbance was measured at an excitation of 500–530 nm and emission at 590 nm. In the case of cancer tissue, it was finely chopped in the reaction mixture, centrifuged, and then progressed in the same way as cells.

### 2.10. Mice Blood Analysis

Blood parameters (aspartate aminotransferase (AST), alanine aminotransferase (ALT), gamma-glutamyl transferase (GGT), and creatinine (CREA)) were analyzed using a Samsung LABGEOPT Biochemistry Test 9 Kit (PR-PT05) with a Samsung LABGEO PT10 Analyzer according to the manufacturer's instructions.

### 2.11. Statistical Analysis

All statistical analyses were performed using GraphPad Prism 8 software (GraphPad Software, La Jolla, CA, USA), and differences between groups were evaluated using *t*-tests. For multiple comparisons, a one-way analysis of variance was performed, and Tukey's tests were carried out for post hoc analyses. Data are presented as the mean ± standard error of the mean. *P* ≤ 0.05 was considered statistically significant. All data are representative of at least three independent experiments.

## 3. Results

### 3.1. Sericite Inhibits Cell Proliferation by Inducing Cell Cycle Arrest and Apoptosis in MDA-MB231 Cells

**Figure 1:**
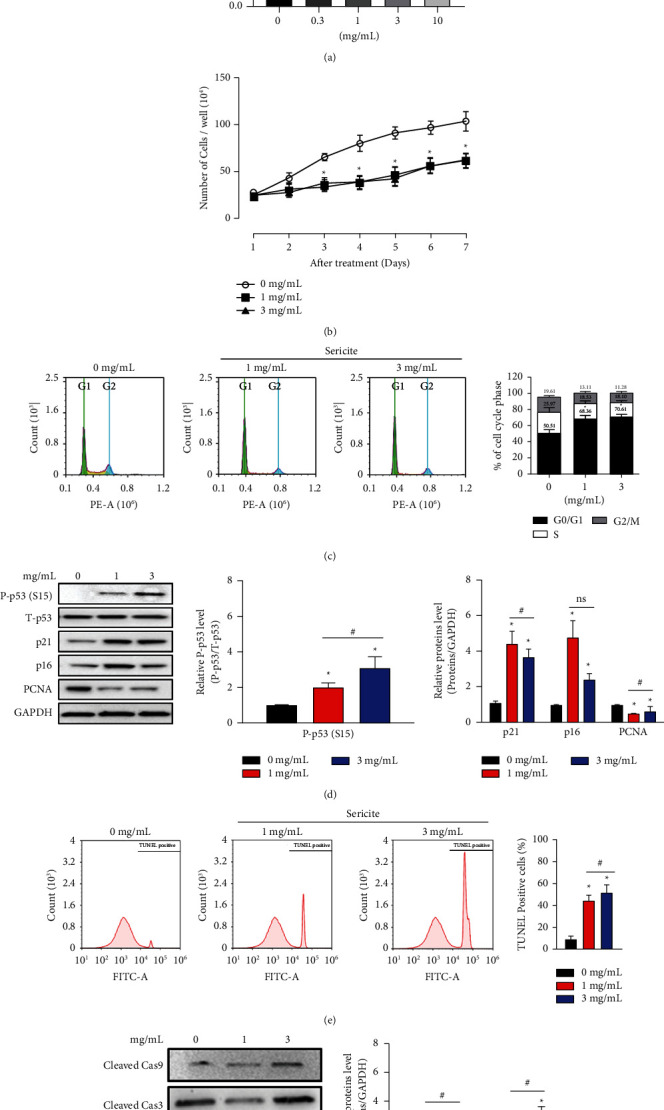
Sericite inhibits cell proliferation by inducing cell cycle arrest and apoptosis in MDA-MB231 cells. MDA-MB231 cells were treated with different concentrations of sericite for 24 h. (a) Cell proliferation assay was performed using a CCK-8 kit. (b) Cell number was measured using an ADAM-MC cell counting machine during 7 days of sericite treatment. (c) Cell number was detected using fluorescence-activated cell sorting (FACS) analysis. (d) Cell cycle arrest-related proteins were detected by immunoblotting. (e) The apoptotic rare in MDA-MB231 cells was measured by TUNEL staining after sericite treatment. (f) Apoptotic proteins were detected by immunoblotting. GAPDH was used as an internal control. The protein levels were qualified by densitometric analysis (right panel). All data are representative of three independent experiments. Data are presented as mean ± SEM of three independent experiments. ^*∗*^*P* < 0.05 vs. 0 mg/mL sericite-treated MDA-MB231 cells. ^#^*P* < 0.05 vs. 1 mg/mL sericite-treated MDA-MB231 cells.

We first investigated whether sericite could suppress the proliferation of human breast cancer cell lines BT474 (triple positive), MCF7 (ER and PR positive and HER2 negative), SKBR3 (ER and PR negative and HER2 positive), BT549 and MDA-MB231 (triple-negative), and MCF10A (normal breast epithelial cells). After treatment with sericite at different concentrations for 24 h, a CCK-8 cell proliferation assay was performed. The sericite-induced decrease in cell proliferation was more effective in MDA-MB231 cells than other breast cancer cells (Figure 1(a) and Supplementary Figure 1). We chose MDA-MB231 cells and sericite concentrations of 1 mg/mL and 3 mg/mL for further investigation of anticancer mechanisms.  Figure 1(b) and Supplementary Figure 2A show that sericite treatment decreased cell growth; therefore, the number of cells after sericite treatment was significantly lower than those untreated cells and the cells treated with sericite slowly started showing features of apoptosis. Sericite arrested cells in the G0/G1 phase of the cell cycle (Figure 1(c)), increased the levels of the cell cycle regulatory proteins: p21, p16, and phosphorylation of p53 at its serine 15 site (S15), which is the primary target of DNA damage response on the p53 protein, while decreasing the level of PCNA (Figure 1(d)). Furthermore, to determine the mechanism by which sericite treatment induces apoptosis in MDA-MB231 cells, we examined DNA damage using a terminal deoxynucleotidyl transferase (TdT) dUTP Nick end labeling (TUNEL) assay and fluorescence microscopy (Figure 1(e) and Supplementary Figure 2B). Data shows that sericite treatment induced DNA fragmentation in MDA-MB231 cells, a hallmark of apoptosis. We also looked for changes in apoptotic protein markers and found a significant induction of cleaved caspase-9 and increased activation of caspase-3 cleavage, which activated apoptosis in sericite-treated cells (Figure 1(f)).

### 3.2. Sericite Downregulates HSPs and ROS in MDA-MB231 Cells

**Figure 2:**
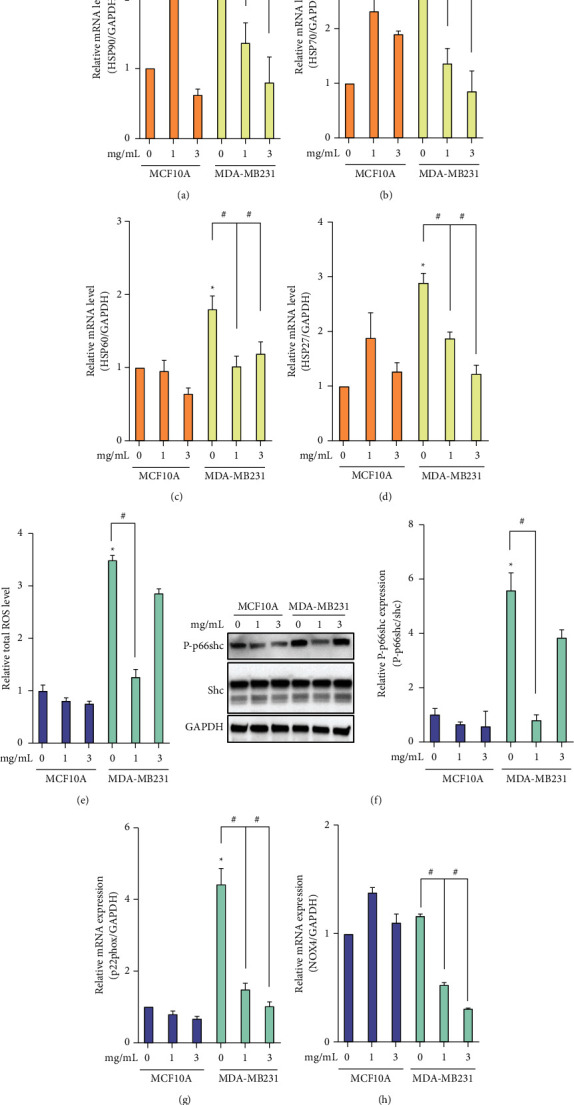
Sericite regulated HSPs and ROS in MDA-MB231 cells. MDA-MB231 cells were treated with different concentrations of sericite for 24h. (a–d) mRNA expressions were detected using an Amplex Red assay. (f) Phosphorylation of p66shc was measured by immunoblotting. (g, h) mRNA expressions of p22phox and NOX4 were detected by qPCR. GAPDH was used as an internal control for both immunoblotting and qPCR. All data are representative of three independent experiments. Data are presented as mean ± SEM of three independent experiments. ^*∗*^*P* < 0.05 vs. 0 mg/mL sericite-treated MDA-MB231 cells. ^#^*P* < 0.05 vs. 1 mg/mL sericite-treated MDA-MB231 cells.

HSPs act as stress proteins, rapidly created after exposure to unfavorable extrinsic factors such as high temperature, hypoxia, or cytokine release. Increased levels of HSP are required by the oncoproteins of cancer for their folding, aggregation, stabilization, function, activation, and proteolytic degradation. The expressions and activities of Hsp27, Hsp70, and Hsp90 chaperones are markedly higher in cancer [13]. We quantified the levels of HSP mRNA in MDA-MB231 cells and a normal breast epithelial cell line, MCF10A. HSP90, HSP70, HSP60, and HSP27 were overexpressed in MDA-MB231 compared to MCF10A cells; however, this overexpression was reduced by sericite treatment (Figures 2(a)–2(d)). These data demonstrate that overexpression of HSPs strengthens cancer cell proliferation and that sericite attenuates this proliferation via a reduction in HSP expression.

Generally, the expression of ROS in cancer cells is higher than in normal cells. ROS can promote protumorigenic signaling and facilitate cancer cell proliferation, survival, and adaptation to hypoxia [14]. Therefore, reducing the ROS levels to impede their role in the cellular transformation of cancer cells could be a possible ROS-related anticancer therapeutic strategy. We first confirmed that ROS were significantly upregulated in MDA-MB231 cells compared to MCF10A and found that sericite treatment suppressed the total ROS level in MDA-MB231 cells as measured by the Amplex Red assay (Figure 2(e)). Phosphorylation of p66shc is known as a key regulator of ROS metabolism involved in aging and several diseases [15]. We found that sericite treatment reduced p66shc phosphorylation in both MDA-MB231 and MCF10A cells (Figure 2(f)). As an oxygen sensor, the NADPH oxidase 4 (NOX4)/p22phox enzymatic complex plays a diverse role in cell proliferation, migration, and cell death. Increased expression of the NOX4/p22phox complex in cancer has been previously reported, which activates angiogenesis and metastasis [16]. Sericite treatment effectively attenuated p22phox and NOX4 mRNA expression in MDA-MB231 cells (Figures 2(g) and 2(h)).

### 3.3. Sericite Suppresses Cancer Growth in an MDA-MB231 Xenograft Mouse Model

**Figure 3:**
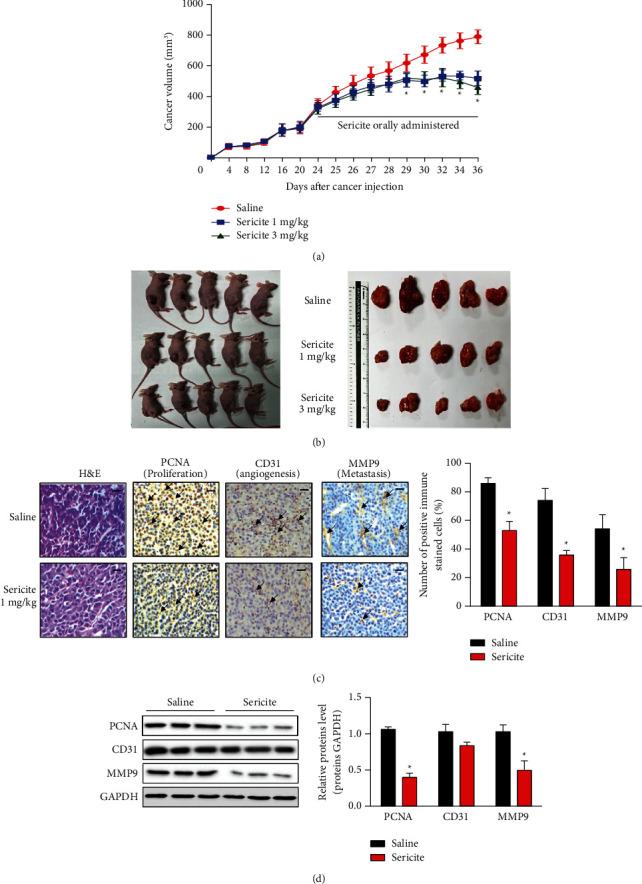
Sericite suppresses cell proliferation in an MDA-MB231 xenograft mouse model. MDA-MB231 cells were xenotransplanted into mice followed by a 24-day stabilization period, after which sericite was orally administrated daily in two different doses (1 mg/kg or 3 mg/kg) for 12 days. Tumor size was measured daily. (a) The 12-day tumor volume measurements. (b) Photographs of mice bearing tumors and tumors after extraction from the mice. (c) Isolated tumors were stained with H and E for PCNA, CD31, and MMP9. Scale bar, 50 *μ*m. Arrows indicate positive DAB staining. (d) PCNA, CD31, and MMP9 protein expression were detected by immunoblotting. All data are representative of three independent experiments. Data are presented as mean ± SEM of three independent experiments. ^*∗*^*P* < 0.05 vs. sericite-fed mice.

Over a period of 36 days, the cancer volume (length *∗* width^2^/2) of the control group grew from 400 mm^3^ to 900 mm^3^. However, the sericite-treated groups demonstrated reduced cancer growth, with mean cancer volumes only reaching 600 mm^3^ in the same period (Figures 3(a) and 3(b)). Furthermore, there was no significant change in the weight of the mice until the end of the experiment (Supplementary Figure 3A). To investigate any side effects of sericite treatment in the mice, we performed blood analysis of control and sericite-treated mice to analyze the kidney and liver functions based on the measurement of aspartate aminotransferase (AST), alanine aminotransferase (ALT), and gamma-glutamyl transpeptidase (GGT) for the liver function, and creatinine (CREA) for the kidney function. No significant difference was found between the two groups, and the values were in the normal range (Supplementary Figure 3B). We chose a 1 mg/kg sericite concentration to investigate possible anticancer mechanisms *in vivo*. Tumor paraffin sections from saline-fed mice and 1 mg/kg sericite-fed mice were subjected to hematoxylin and eosin (H&E) staining, which revealed compact and loose epithelial cells in the saline and sericite-fed groups, respectively (Figure 3(c)). Proliferating cell nuclear antigen (PCNA) is a marker of cell proliferation in various cancers. We stained tumor sections using anti-PCNA antibodies and found that the sericite group showed lower proliferation (brown) compared to tumor sections from the saline group (Figure 3(c)). Neovascularization is the formation of new blood vessels originating from the endothelium of existing vasculature. Tumor sections from sericite-fed mice exhibited less neovascularization (CD31, brown) and a lower metastatic index (MMP-9, brown) than those from the saline group (Figure 3(c)). We also examined the protein expressions of PCNA, CD31, and MMP9 and found similar results (Figure 3(d)). Taken together, our results suggest that sericite treatment inhibits MDA-MB231 cell proliferation, neovascularization, and metastasis.

### 3.4. Sericite Downregulates HSP and ROS Levels in an MDA-MB231 Xenograft Mouse Model

**Figure 4:**
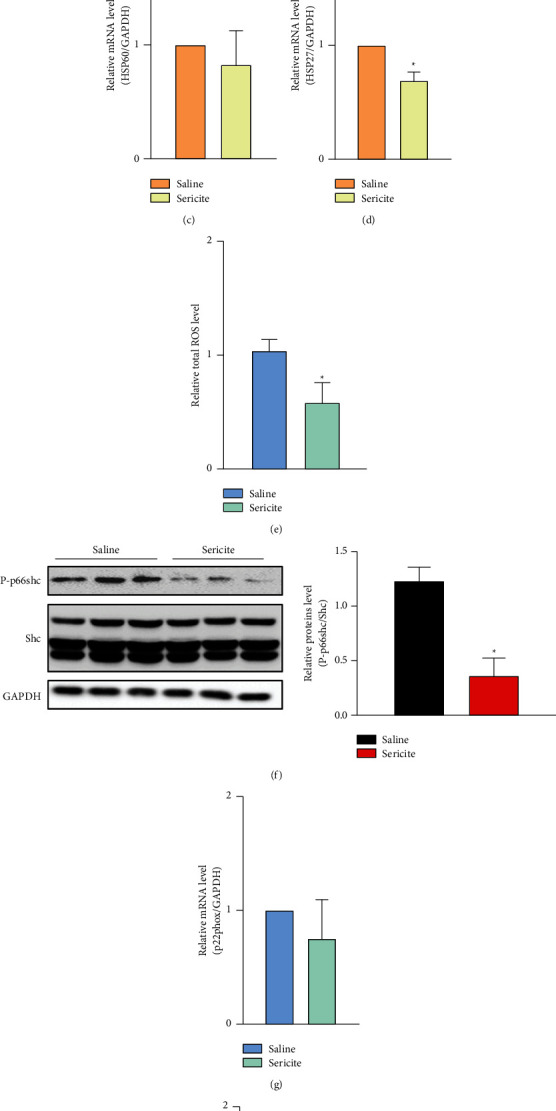
Sericite attenuates HSP- and ROS-related pathways in an MDA-MB231 xenograft mouse model. (a–d) mRNA expressions of HSPs in saline-fed mice and 1 mg/kg sericite-fed mice. (e) Intracellular ROS was detected using an Amplex Red assay. (f) Phosphorylation of p66shc was measured by immunoblotting. (g and h) mRNA expressions of p22phox and NOX4 were detected by qPCR. All data are representative of three independent experiments. Data are presented as mean ± SEM of three independent experiments. ^*∗*^*P* < 0.05 vs. sericite-fed mice.

Since we found that sericite treatment suppressed the proliferation of MDA-MB231 cells, we investigated the effects of sericite on cell protection *in vivo*. After sericite treatment, the mRNA levels of HSP90, HSP70, and HSP27 were attenuated in the MDA-MB231 xenograft model (Figures 4(a)–4(d)). We also observed that intracellular ROS was significantly decreased in sericite-treated mouse neoplasms (Figure 4(e)), along with reduced phosphorylation of p66shc (Figure 4(f)) and reduced mRNA expression of p22phox (Figure 4(g)) and NOX4 (Figure 4(h)), which are upstream regulators of p66shc. These findings suggest that sericite reduces the growth of MDA-MB231 cells *in vivo* by suppressing HSPs and ROS.

## 4. Discussion

TNBC demonstrates higher malignancy, lower survival rates, frequent relapses, and higher mortality rates among all breast cancer subtypes. Moreover, TNBC incidence is more frequent and prevalent in younger patients than in elderly patients [17, 18]. Therefore, young women are in need of novel TNBC treatments, and a better understanding of the molecular mechanisms responsible for cancer metastasis may provide a vision to improve TNBC patient survival. In the present study, we investigated antitumor effects of sericite against MDA-MB231 breast cancer cells. Sericite administration effectively suppressed tumor growth of MDA-MB231 cells in a xenograft mouse model. Previous studies have provided evidence that minerals have an antitumor effect in breast cancer cells [19, 20]. Most of these studies found that treating cancer cells with minerals like mica inhibits cell growth or induces apoptosis by the regulation of crucial receptors or signaling pathways. Our results are consistent with these studies and support the notion that sericite inhibits cell growth and proliferation and induces apoptosis in MDA-MB231 cells.

Evidence suggests that minerals have antitumor activity in several types of cancer. For instance, selenium, arsenic trioxide, zinc, and cadmium have been reported to have antitumor effects in various cancers, including breast cancer [8]. Similarly, mica particles have been studied to discern their function and to find the most effective formulation for oral supplements. The immune-enhancing effects of mica in animals have been evaluated as a dietary aluminosilicate supplement (DAS) in mice. A DAS showed clearance effects on pig circovirus type 2 in experimentally infected pigs, and mild side effects in mice and pigs were observed during the daily administration of the DAS [21].

In the present study, we first investigated the *in vitro* effects of sericite in MDA-MB231 cells. Sericite treatment decreased cell proliferation and cell number in a concentration-dependent manner. A reduction in cell proliferation has been proven to be an effective tumor-suppressing mechanism. Previous studies have shown that celecoxib (CBX), which is a nonsteroidal anti-inflammatory drug (NSAIDs), and a potent COX-2 selective inhibitor suppresses cell proliferation in vitro and in vivo by mechanisms that include inhibition of cell cycle [22]. Similarly, sericite may inhibit proliferation by inducing a growth arrest in tumor cells. We demonstrated that sericite treatment increased the G0/G1 cell population in a concentration-dependent manner and enhanced the expression of the cell cycle arrest markers P53, P21, and P16. Some bioactive ingredients such as thymoquinone (TQ) and costunolide (COS) have been reported to promote apoptosis in human colon and breast cancer cell lines via a p53 dependence. Similarly, doxorubicin (DOX), a chemotherapy drug, induces upregulation of phosphorylation of p53 at S15, consequently upregulating p21 and inducing cell cycle arrest [23]. Furthermore, the present study investigated the mechanism responsible for sericite-mediated cytotoxicity. Promoting tumor cell apoptosis is one of the most important methods of tumor treatment. Induction of apoptosis in cancer cells by treatment with CBX has been documented earlier in gastric cancer cell lines [22]. The results of our TUNEL assay revealed that sericite-treated cancer cells underwent apoptosis. This fact was supported by a western blot analysis of the apoptosis markers caspase-9 and caspase-3, which were found to be upregulated in sericite-treated cells confirming the apoptotic effect.

HSPs are ubiquitously found in all living organisms and their expression is induced/regulated by stress. Previously, it was thought that HSPs are induced by heat alone; however, it is now known that various types of physiological or pathological stresses may regulate their expression [24]. The HSPs expression is increased during oncogenesis, resulting in malignant transformation and promoting rapid somatic evolution. Recently, HSPs are evolving as molecular targets in cancer therapy by the interference of their diverse functions in cancer cells following different approaches. There are clinical trials for various cancers, including breast cancer, using HSP-inhibitor compounds, and other HSP-based strategies [25, 26].

Similarly, high levels of ROS have been detected in cancer cells due to various changes like increased receptor signaling, increased metabolic and peroxisomal activities, mitochondrial dysfunction, and oncogenic activity [27]. Previous studies have revealed that various prospective compounds, namely quercetin, metformin, vitamin C, and curcumin, have been found to downregulate ROS in the cellular apoptotic process. Furthermore, some have been shown to promote apoptosis in cancer cells [28]. *Shc* gene regulates the level of ROS, apoptosis induction, and lifespan in mammals. *Shc* is a *Src* homology 2 domain-containing protein and is a member of an adaptor family of proteins. Shc has 3 isoforms based on the molecular weight of 46, 52, and 66 kDa (p46shc, p52shc, and p66shc) [29]. p66shc is the longest isoform with an additional CH2 domain containing a S36 residue which is phosphorylated in response to oxidative stress along with a role in apoptosis [30]. p46shc and p52shc are universally expressed; however, p66shc is expressed at different levels in different tissues [31]. p66shc is part of a signal transduction pathway activated in response to increased ROS. It is phosphorylated on ser36 after exposure to oxidative stress from H_2_O_2_ or ultraviolet light, and this phosphorylation is critical for the cell death response evoked by oxidative damage [32]. In this study, sericite treatment reduced the levels of HSPs, ROS, and p66shc both *in vitro* and *in vivo*. Furthermore, sericite treatment attenuated the expression of the survival gene PCNA, reduced the typical angiogenesis marker CD31, and reduced the expression of the metastasis marker MMP9 in tumor tissues, indicating the antiproliferative, antiangiogenic, and antimetastatic activity of sericite in a xenograft model along with a reduction in tumor volume. One of the limitations of this study is the poor solubility of sericite powder in the cell culture media and in saline for the in vitro and in vivo experiments, respectively. Consequently, the concentration range used in this study was limited, and future work will be required to solve this problem by finding new dilution methods or ways to improve sericite solubility.

## 5. Conclusion

In conclusion, these findings suggest that sericite has chemopreventive potential in TNBC, which could be a basis for developing alternative therapies to treat tumor cells using natural compounds.

## Data Availability

The data used to support the findings of this study are available from the corresponding author upon request.

## Supplementary Materials

Supplementary Materials
*Supplementary Fig.1*. Effect of sericite treatment on cell proliferation of different breast cancer cell lines. (A) MCF10A, (B) BT474, (C) MCF7, (D) SKBR3, and (E) BT549 cells were treated with different concentrations of sericite for 24 h, followed by detection of cell proliferation using CCK-8 assay. Data are presented as the mean ± SEM of three independent experiments. ^*∗*^*P* < 0.05 vs. 0 mg/mL sericite-treated cells*. Supplementary Fig.2.* Effect of sericite treatment on cell morphology and apoptosis in MDA-MB231 cells. (A) Effect of sericite treatment on cell morphology up to 7 days of incubation. (B) Images show merged staining with Propidium Iodide/RNase A (red) and FITC-dUTP (green) (TUNEL) after sericite treatment in MDA-MB231 cells. Scale bar = 20 *μ*m. Data are presented as the mean ± SEM of three independent experiments. *Supplementary Fig.3.* Effect of sericite treatment on the health of mice. (A) Changes in animal body weight. (B) Blood analysis of mice was performed to analyze the kidney and liver function. Aspartate aminotransferase (AST), alanine aminotransferase (ALT), and gamma-glutamyl transpeptidase (GGT) are a measure of liver function. Creatinine (CREA) is the measure of kidney function. The values of all the components were in the normal range (AST: 0–40 U/L, ALT: 0–45 U/L, GGT: 0–66 U/L, and CREA: 0.5–1.3 mg/dl). Data are presented as the mean ± SEM of five independent experiments. *Table S1.* Information on qPCR primers. *Supplementary Fig.5–Supplementary Fig.9.* Full-length western blot gel images.
