# Supplementary material for: Antitumor Potential of Sericite Treatment Mediated by Cell Cycle Arrest in Triple-Negative MDA-MB231 Breast Cancer Cells
Source: Evid Based Complement Alternat Med. 2022 Sep 25;2022:2885293. doi: 10.1155/2022/2885293 (PMC9527418; doi:10.1155/2022/2885293)
Supplement: Supplementary Materials — Supplementary Fig.1. Effect of sericite treatment on cell proliferation of different breast cancer cell lines. (A) MCF10A, (B) BT474, (C) MCF7, (D) SKBR3, and (E) BT549 cells were treated with different concentrations of sericite for 24 h, followed by detection of cell proliferation using CCK-8 assay. Data are presented as the mean ± SEM of three independent experiments. ∗P < 0.05 vs. 0 mg/mL sericite-treated cells. Supplementary Fig.2. Effect of sericite treatment on cell morphology and apoptosis in MDA-MB231 cells. (A) Effect of sericite treatment on cell morphology up to 7 days of incubation. (B) Images show merged staining with Propidium Iodide/RNase A (red) and FITC-dUTP (green) (TUNEL) after sericite treatment in MDA-MB231 cells. Scale bar = 20 μm. Data are presented as the mean ± SEM of three independent experiments. Supplementary Fig.3. Effect of sericite treatment on the health of mice. (A) Changes in animal body weight. (B) Blood analysis of mice was performed to analyze the kidney and liver function. Aspartate aminotransferase (AST), alanine aminotransferase (ALT), and gamma-glutamyl transpeptidase (GGT) are a measure of liver function. Creatinine (CREA) is the measure of kidney function. The values of all the components were in the normal range (AST: 0–40 U/L, ALT: 0–45 U/L, GGT: 0–66 U/L, and CREA: 0.5–1.3 mg/dl). Data are presented as the mean ± SEM of five independent experiments. Table S1. Information on qPCR primers. Supplementary Fig.5–Supplementary Fig.9. Full-length western blot gel images. [file 2885293.f1.pdf]

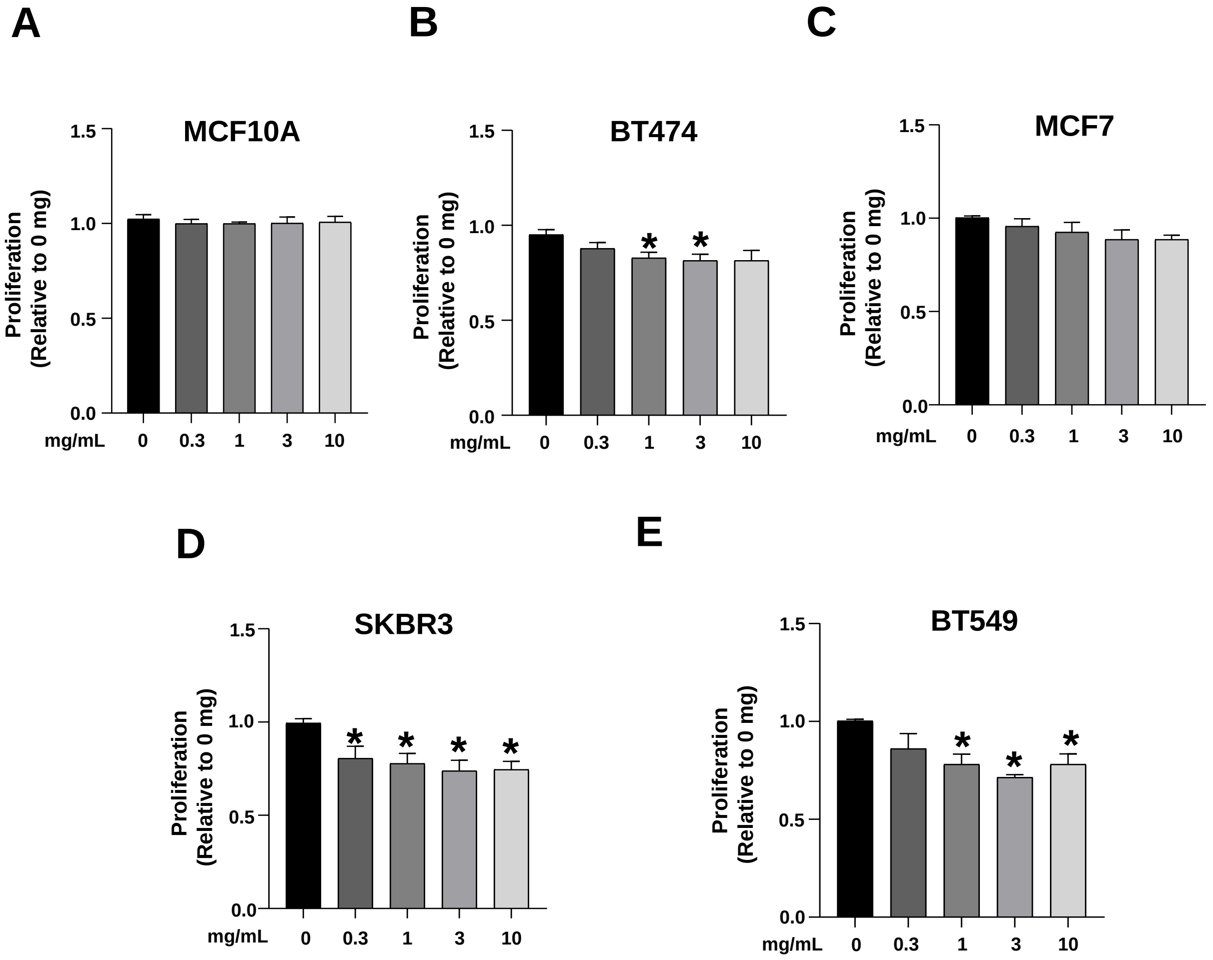

Supplementary Fig.1. Effect of Sericite treatment on cell proliferation of different breast cancer cell lines.

(A) MCF10A (B) BT474 (C) MCF7 (D) SKBR3 and (E) BT549 cells were treated with different concentrations of sericite for 24 h followed by detection of cell proliferation using CCK-8 assay. Data are presented as the mean ± SEM of three independent experiments. *P < 0.05 vs. 0 mg/mL sericite-treated cells.

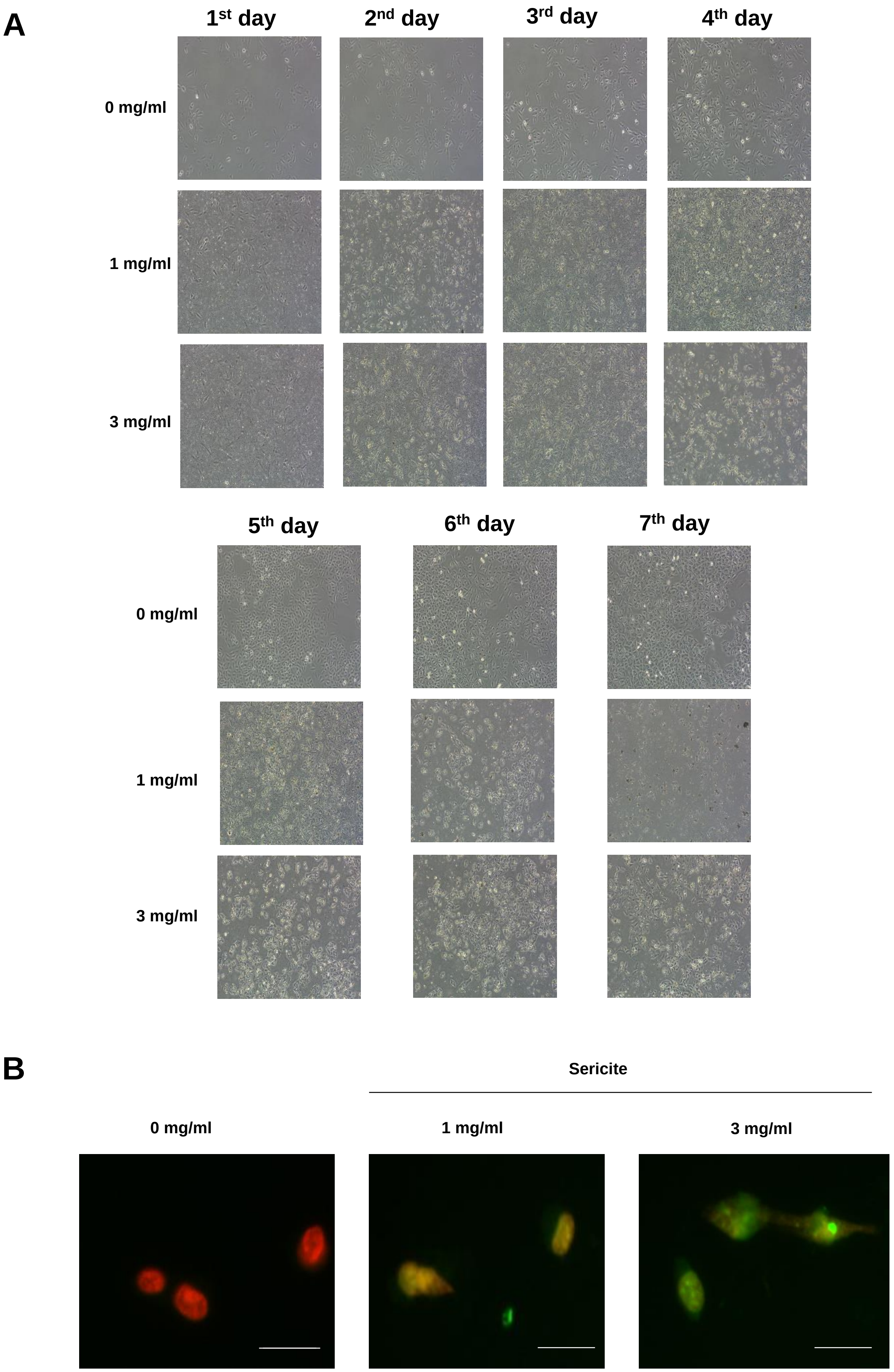

Supplementary Fig.2. Effect of Sericite treatment on cell morphology and apoptosis in MDA-MB231 cells.

(A) Effect of sericite treatment on cell morphology up to 7 days of incubation. (B) Images show merged staining with Propidium Iodide/RNase A (red) and FITC-dUTP (green) (TUNEL) after sericite treatment in MDA-MB231 cells. Scale bar = 20 μm. Data are presented as the mean ± SEM of three independent experiments.

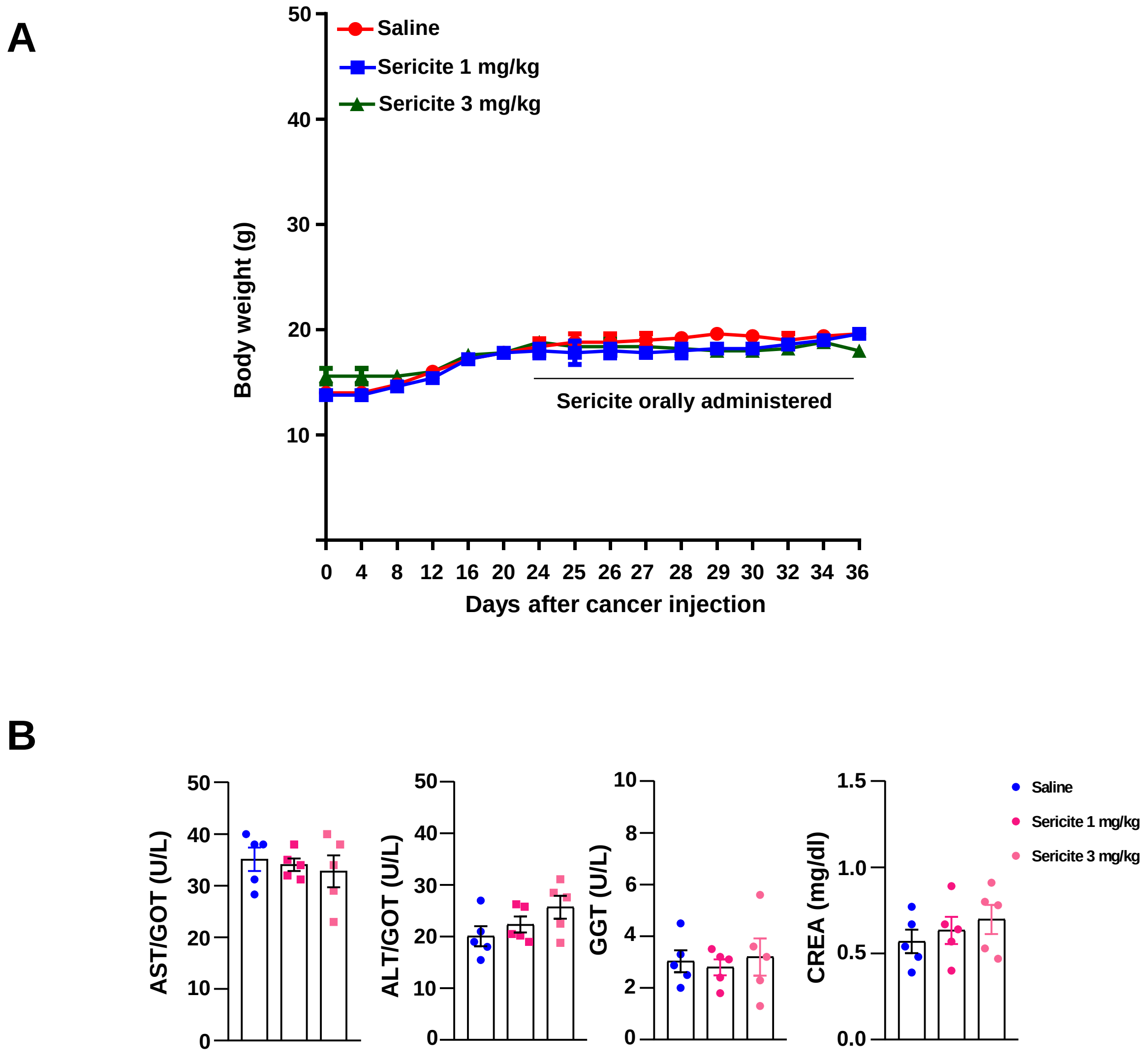

Supplementary Fig.3. Effect of Sericite treatment on the health of mice.

(A) Changes in animal body weight. (B) Blood analysis of mice was performed to analyze the kidney and liver function. Aspartate aminotransferase (AST), Alanine aminotransferase (ALT) and Gamma-glutamyl transpeptidase (GGT) are a measure of liver function. Creatinine (CREA) is the measure of kidney function. The values of all the components were in the normal range (AST: 0-40 U/L, ALT: 0-45 U/L, GGT: 0-66 U/L and CREA: 0.5-1.3 mg/dl). Data are presented as the mean ± SEM of five independent experiments.

Primer	Forward	Reverse	Accession Num.
HSP90	5'-GGG CTT CGA GGT GGT GTA TAT-3'	5'-TGC CTT GCT CTC CTC CAT TTT-3'	NM_001017963.3
HSP70	5'-AGG TGA ACT ACA AGG GCG AGA-3'	5'-ATG ATC CGC AGC ACG TTT AGA-3'	NM_005345.6
HSP60	5'-AGT CCA TTG TCC CTG CTC TTG-3'	5'-TGA CTG CCA CAA CCT GAA GAC-3'	NM_199440.2
HSP27	5'-GAG AGT TCT GTC GCA CCT ATG-3'	5'-AGC AGG AAG CAG GGA GAT GAT GTA-3'	AB020027.1
NOX4	5'-GCA GGA GAA CCA GGA GAT TG-3'	5'-CAC TGA GAA GTT GAG GGC ATT-3'	NM_016931.5
p22phox	5'-CGC TGG CGT CCG CCT GAT CCT CA-3'	5'-ACG CAC AGC CGC CAG TAG GTA GAT-3'	NM_000101.4
GAPDH	5'-ATG GGG AAG GTG AAG GTC G-3'	5'-GGG GTC ATT GAT GGC AAC AAT A	NM_001357943.2

Table S1. **Information of qPCR primers**

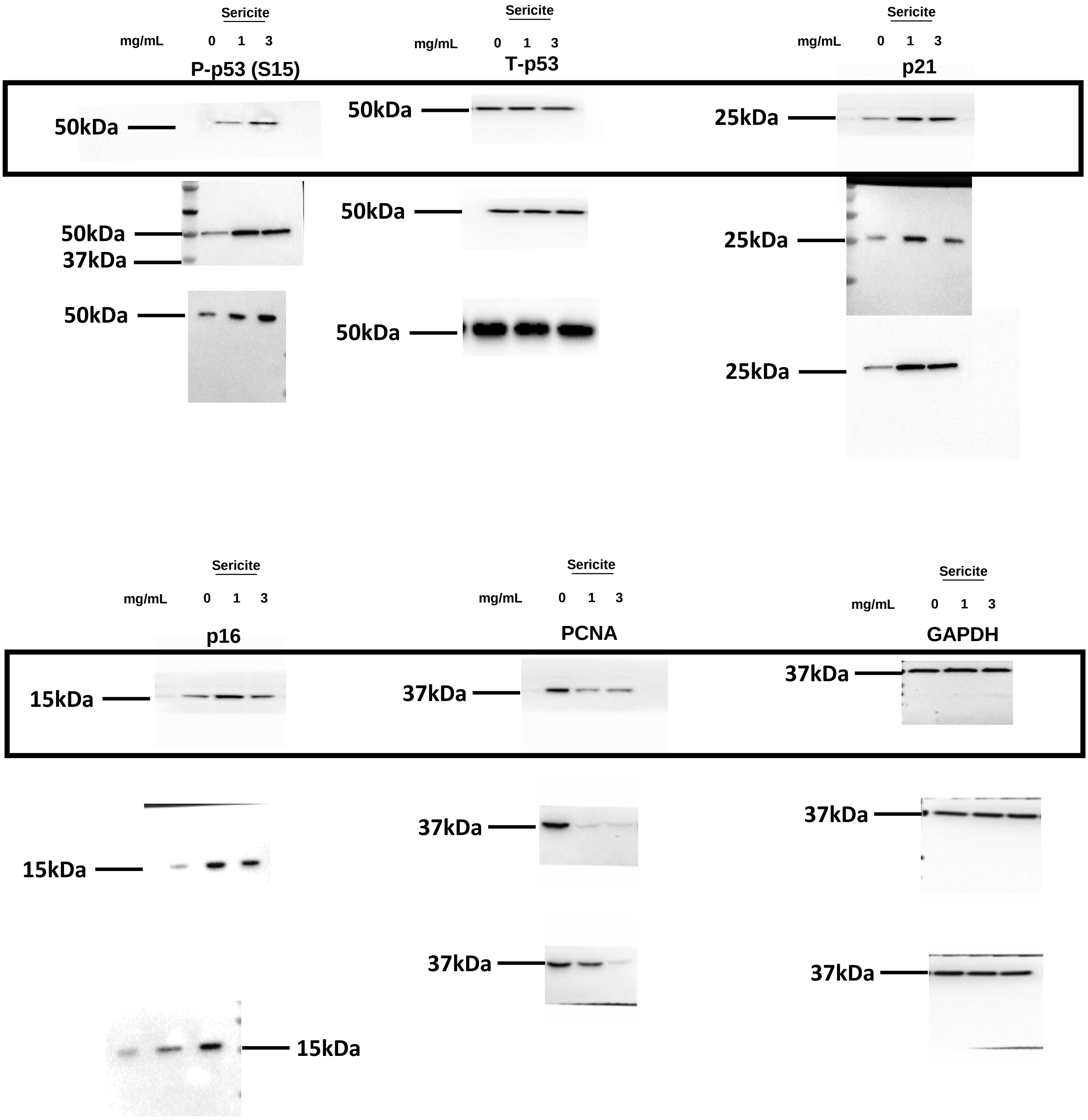

Supplementary Fig.5. Full-length western blot gel image for figure 1D.

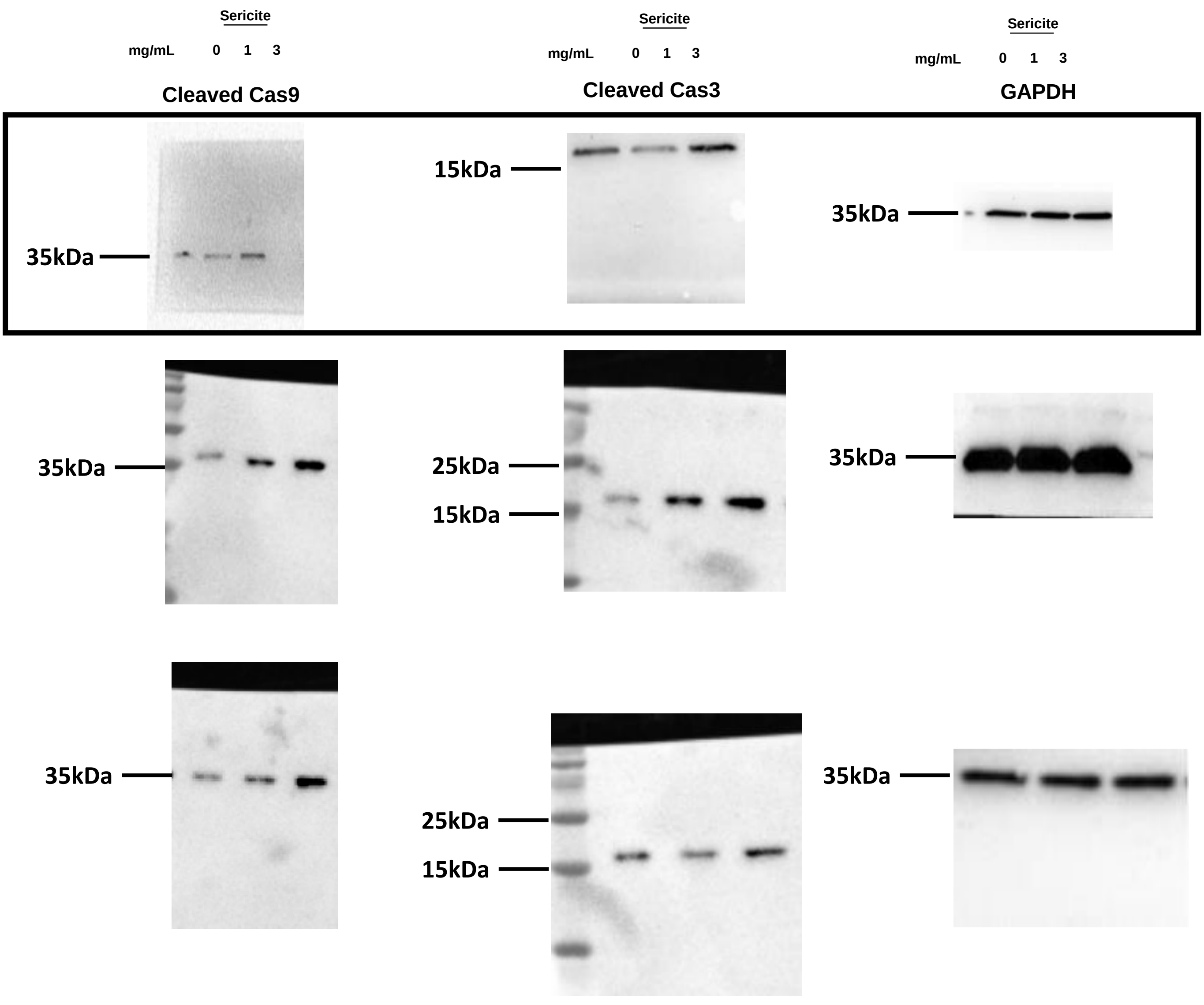

Supplementary Fig.6. Full-length western blot gel image for figure 1F.

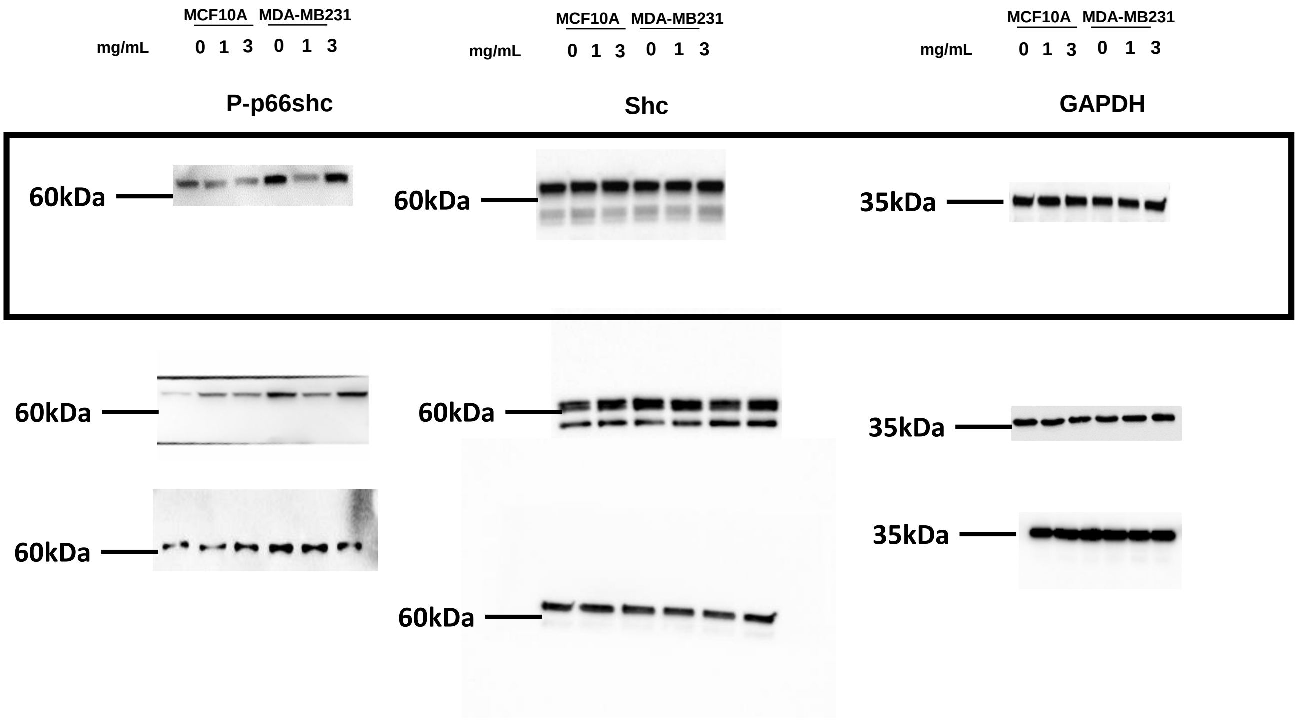

Supplementary Fig.7. Full-length western blot gel image for figure 2F.

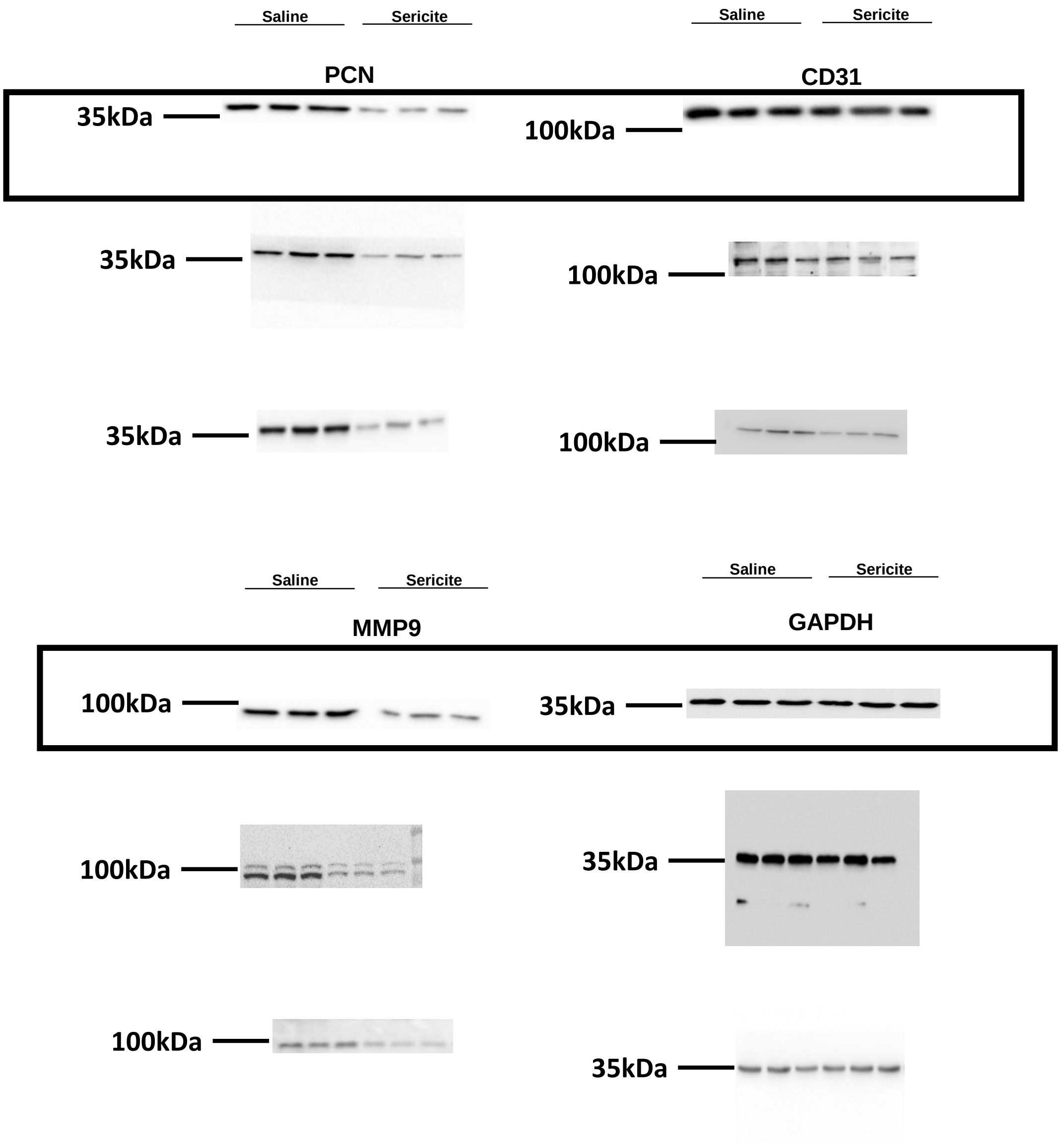

Supplementary Fig.8. Full-length western blot gel image for figure 3D.

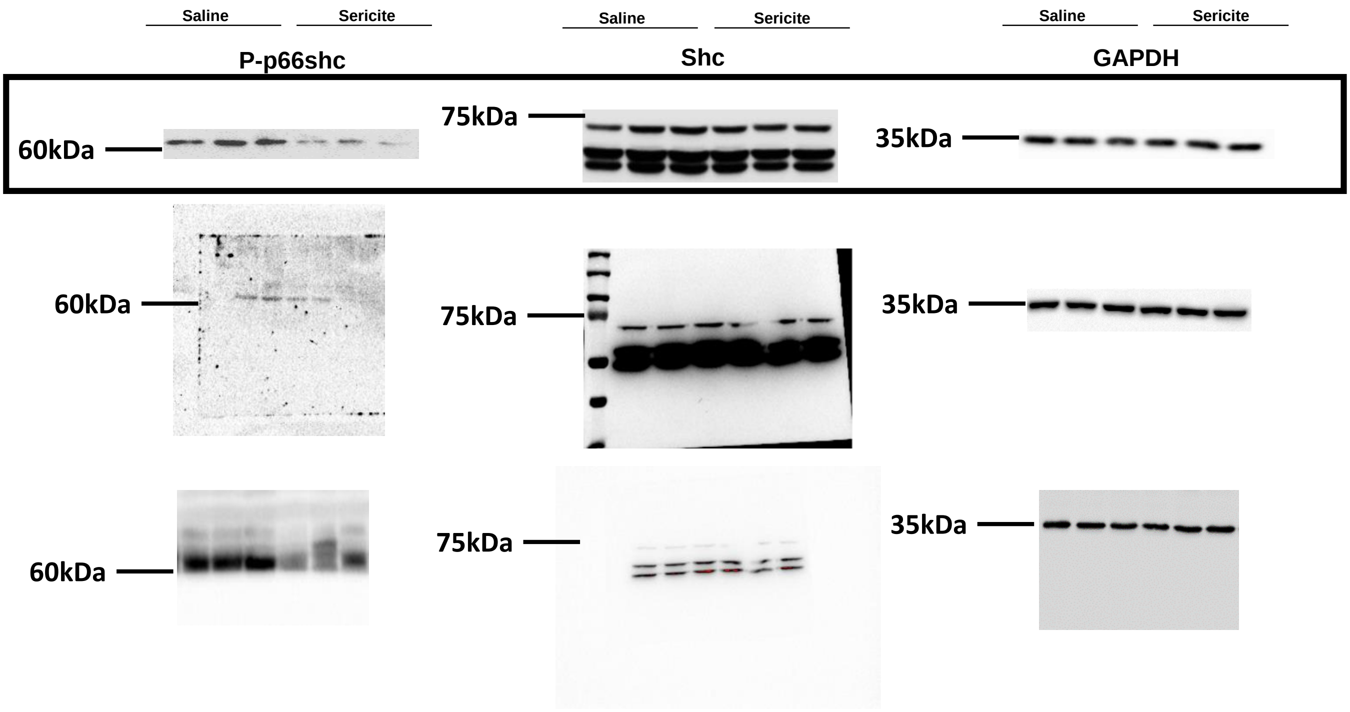

Supplementary Fig.9. Full-length western blot gel image for figure 4F.
